# Exploring the Potential of Cotton Industry Byproducts in the Plastic Composite Sector: Macro and Micromechanics Study of the Flexural Modulus

**DOI:** 10.3390/ma14174787

**Published:** 2021-08-24

**Authors:** Albert Serra, Ferran Serra-Parareda, Fabiola Vilaseca, Marc Delgado-Aguilar, Francesc X. Espinach, Quim Tarrés

**Affiliations:** 1 LEPAMAP-PRODIS Research Group, University of Girona, Maria Aurèlia Capmany 61, 17003 Girona, Spain; albert.serra@udg.edu (A.S.); ferran.serrap@udg.edu (F.S.-P.); m.delgado@udg.edu (M.D.-A.); francisco.espinach@udg.edu (F.X.E.); 2 Advanced Biomaterials and Nanotechnology, Department of Chemical Engineering, University of Girona, Maria Aurèlia Capmany 61, 17003 Girona, Spain; fabiola.vilaseca@udg.edu; 3 Chair on Sustainable Industrial Processes, University of Girona, Maria Aurèlia Capmany 61, 17003 Girona, Spain

**Keywords:** cotton fibers, textile byproduct, flexural modulus, composites, circular economy

## Abstract

The textile sector produces yearly great quantities of cotton byproducts, and the major part is either incinerated or landfilled, resulting in serious environmental risks. The use of such byproducts in the composite sector presents an attractive opportunity to valorize the residue, reduce its environmental impact, and decrease the pressure on natural and synthetic resources. In this work, composite materials based on polypropylene and dyed cotton byproducts from the textile industry were manufactured. The competitiveness of the resulting composites was evaluated from the analyses, at macro and micro scales, of the flexural modulus. It was observed that the presence of dyes in cotton fibers, also a byproduct from the production of denim items, notably favored the dispersion of the phases in comparison with other cellulose-rich fibers. Further, the presence of a coupling agent, in this case, maleic anhydride grafted polypropylene, enhanced the interfacial adhesion of the composite. As a result, the flexural modulus of the composite at 50 wt.% of cotton fibers enhanced by 272% the modulus of the matrix. From the micromechanics analysis, using the Hirsch model, the intrinsic flexural modulus of cotton fibers was set at 20.9 GPa. Other relevant micromechanics factors were studied to evaluate the contribution and efficiency of the fibers to the flexural modulus of the composite. Overall, the work sheds light on the potential of cotton industry byproducts to contribute to a circular economy.

## 1. Introduction

The circular economy has recently evolved and gained acceptance mainly due to the increasing environmental awareness in our society, scarcity of resources, and environmental legislation [1,2]. One major goal of the circular economy is the minimization of waste generation in industrial processes, or otherwise, the implementation of systems that promote conscientious management of such wastes via, for example, its valorization in other sectors [3]. In this context, the textile sector has experienced considerable growth in recent years, reaching up to approximately 105 million tons per year of textile fibers by 2018 [4], which by consequence has driven the generation of high amounts of textile byproducts [5]. A large fraction of such textile byproducts with low-added value are landfilled or incinerated, resulting in serious environmental risks, whilst only a minor part is recycled. As reported, only 15% of the textile byproducts worldwide are currently recycled or reused, and it is estimated that nowadays such wastes occupy 5% of the mass of landfills [6]. To enhance the sustainability of the textile sector and reduce the environmental impact resulting from the improper waste management strategies, the application of a circular economy and valorization of the byproducts is of utmost importance.

It is estimated that a great part, about 35–40%, of the textile waste produced globally consists of cotton [7,8]. The production of cotton fabrics consists of a first step where the cotton fibers are yarned to produce high-quality yarns, which are then destined to the manufacturing of fabrics and the obtention of textiles. This step generates high content of fibrous residues in the shape of cotton trims, which are posteriorly submitted to a defibration process to obtain cotton fibers. Again, these fibers are yarned for the final production of textiles that will be used for the manufacturing of denim products. These fibers used to produce denim items are generally more than 10 mm in length, whereas those fibers less than 10 mm in length are unable to be yarned and thus have no value to the textile industry. Such fibrous-like material stemming from the yarning of cotton trims has usually been referred to, owing to its shape and appearance, as cotton flocks, which are regarded as a lignocellulosic byproduct of the textile industry. Additionally, the dyes applied for the production of denim products remain in such cotton flocks, which indeed add a layer of complexity to its recycling, as huge amounts of water, energy, and reactants may be needed to eliminate the dyes, making these operations economically and environmentally unfeasible [9].

An attractive way to valorize dyed cotton flocks could be by its incorporation in polymer matrixes to provide improved strength and/or stiffness to composite materials. Such composite materials have been typically produced using synthetic fibers (i.e., glass, carbon, or aramid); though, in recent years, there has been an increasing interest in substituting such materials with natural fibers [10,11,12]. This is mainly due to the much more eco-friendly character of natural fibers over synthetic ones due to their biobased, renewable, recyclable, and biodegradable nature [13]. Additionally, mixing natural fibers and plastic materials may contribute to low-weight, non-abrasive, non-toxic, low-cost, and biodegradable properties [14]. Natural fiber composites (NFC) have been traditionally developed using flax, abaca, bamboo, hemp, sisal, and wood, amongst others [15]. However, in practice, any type of lignocellulosic material, such as recycled fibers, side streams from agricultural practices, or even industrial byproducts, can be added to plastics as reinforcement [16]. In this context, the current investigation aimed to incorporate dyed cotton flocks, an industrial textile byproduct, into polypropylene, with the purpose of valorizing the residue, contributing to a circular economy, reducing the pressure on natural resources, and developing competitive composite materials that can replace the existing ones.

The case of cotton flocks is considered particularly interesting given the huge availability of the residue and its favorable chemical composition. Cotton is a cellulose-rich material with high availability of hydroxyls groups at the fibers’ surface [17]. The abundance of hydroxyl groups can aid the development of bonds between the polymeric and lignocellulosic phases under favorable conditions. For instance, in polypropylene (PP)-based composites, with PP probably being the most representative polyolefin within the composite sector, the use of maleic anhydride grafted polypropylene (MAPP) as a coupling agent has effectively proved to enhance the interfacial adhesion by connecting the hydroxyl groups in the fiber surface and maleic acid chains. The action of MAPP is based on two mechanisms: first, the maleic groups form covalent bonds through esterification with the hydroxyl groups in the surface of the fiber; second, MAPP’s PP chains diffuse through entanglement (physical interactions) between the unmodified PP chains [18,19,20]. Hence, an elevated presence of hydroxyls groups combined with the action of an adequate coupling agent can develop an optimal scenario to enhance the fiber–plastic compatibility. Otherwise, poor compatibilities may lead to fiber agglomeration, uneven dispersion within the matrix, and low stress-transfer capacity, ultimately hindering the composite’s properties. Strengthening the interfacial adhesion with coupling agents may also contribute to the water barrier properties of the composite material by reducing the gaps between the fiber and the matrix [21]. In previous work from the research team dealing with the tensile and flexural strength of cotton fiber-reinforced PP composites, a 6 wt.% of MAPP concerning the fiber content was established as an optimal percentage of coupling agent to effectively address the issue of the interfacial adhesion. At this MAPP content, the higher tensile and flexural strength increments were obtained concerning the uncoupled composite. For this reason, in this work, composite materials were prepared both without and with a 6 wt.% of MAPP. It must be stated that other grades of natural fiber-reinforced PP composites may require lower MAPP contents. This may be explained by the greater contribution of hydroxyl groups in the case of cotton fibers in comparison with other fiber sources (i.e., wood) [18,22].

As mentioned, cotton flocks may contain dyes resulting from the manufacture of denim products. The presence of dyes in cotton flocks has been reported to increase the hydrophobicity of the fibers, and thus, better dispersion of the phases is expected [9,23]. It is even possible to find some studies where the use of coupling agents was deemed unnecessary due to the presence of dyes acting as a hydrophobic agent of the fiber surface. Indeed, such dyes could also be interfering with the action of the coupling agents by reducing the accessibility to the hydroxyls groups [24]. However, disposing of coupling agents may lead to insufficient strength increments and limit the potential of the composite material. Overall, there seems to be widespread rather ambiguous results on the influence of dyes and coupling agents in cotton flocks-reinforced plastic composites.

The development of novel materials might fill demands that cannot be satisfied with the existing materials. In this sense, it is important to evaluate the potential of such materials in their specific application sectors, which are principally the automotive and building/construction sectors for the case of natural fiber composites. In such sectors, the composites are transformed, mainly by injection molding or extrusion processes, to obtain products such as door panels, roofing sheets, seat backboards, windows, and floor tiles, amongst others. These components principally develop structural or semi-structural functions and hence are typically subjected to bending forces, whereas tensile forces are scarce in comparison. This makes the flexural behavior of composite materials particularly relevant for gauging the potential of these products. Engineers and architects have a particular interest in previewing the materials’ behavior under use conditions. When it comes to mechanical properties, Neagu et al. [25] explained that the most important characteristics of composite materials aiming at structural functions are dimensional stability and stiffness, whereas strength has a less important role. Overall, there seems to be an interest in the flexural behavior and stiffness of composite material, altogether making necessary the study of the flexural modulus as a key parameter for determining the technical viability of any product. Further, modeling the flexural modulus of composite materials via micromechanics analysis can be useful in understanding the fiber reinforcing mechanism, in determining the intrinsic flexural modulus of the fibers, and in evaluating the contribution of the fibers to the flexural modulus of the composite. Further, micromechanics analysis allows relevant characteristics of the fibers to be obtained, making it possible to predict their performance in similar systems. Some well-known micromechanical models include the Hirsch and Tsai-Pagano models, which can be used for the prediction of the intrinsic flexural modulus, the Cox-Krenchel model, the Fukuda model, and the Kawata model, amongst others—all of them contributing to a better understanding of the role of natural fibers in polymer composites. These micromechanics models have offered an effective prediction of the intrinsic properties and behavior of natural fiber composites, as reflected in numerous studies [26,27,28].

The present work examines the flexural moduli of PP-based composites processed using injection molding and charged up with 10–50 wt.% of cotton flocks, with the main purpose of valorizing this residue and reducing the pressure on other types of reinforcements, either synthetic or extracted from natural resources. Further, the influence of the coupling agent, MAPP, and dyes on the composites is evaluated. The competitiveness of the developed composites is assessed from macro- and micromechanics analyses of the flexural moduli, hereby considered a relevant mechanical property. Overall, the current investigation sheds light on the possibility of developing high-performance composite materials through the valorization of cotton byproducts stemming from the textile industry, obtaining relevant, yet unknown, parameters of such residues via micromechanics modeling.

## 2. Materials and Methods

### 2.1. Materials

Dyed cotton flocks were kindly supplied by Fontfilva S.L. (Olot, Girona, Spain). These flocks were a byproduct of their yarning process. As mentioned, the flocks were agglomerates non-individualized fibers of less than 10 mm in length. Polypropylene (PP) (Isplen PP090 62M) was used as a polymeric matrix, and it was supplied by Repsol-YPF (Tarragona, Spain). Maleic anhydride grafted polypropylene (MAPP) (Epolene G3015) from Eastman Chemical Products (San Roque, Spain) was added to the composites as a coupling agent. Decahydronaphthalene (decalin) was acquired from Fischer Scientific (Madrid, Spain) and used to dissolve the PP matrix to recover the fibers from the composite materials.

### 2.2. Methods

#### 2.2.1. Preparation of Composite Materials

Cotton flocks were initially passed through a blade mill equipped with a 1 mm mesh screen to individualize the entangled fibers and ensure size homogeneity. The resulting cotton fibers were mixed with PP in a Brabender Plastograph kinetic mixer by Brabender^®^ (Duisburg, Germany). The composites were prepared at weight ratios of 20/80, 30/70, 40/60, and 50/50 (cotton fibers/PP), and the whole mixing process took place at 185 °C, speed of 80 rpm, and 10 min. For each composite formulation, the process was carried out with and without MAPP to evaluate the influence of the coupling agent. For coupled composites, a 6 wt.% of MAPP concerning fiber content was added during the mixing process. Such MAPP quantity has been previously reported to be adequate to strengthen fiber-matrix interfacial adhesion [9]. The obtained materials were cooled down at room temperature and then pelletized using a knives mill equipped with a 5 mm mesh screen. The pellets were stored at 80 °C for 24 h to prevent moisture uptake before further processing.

#### 2.2.2. Obtention of Standard Specimens and Flexural Test

The specimens for the flexural test were produced with an injection molding machine Meteor-40 (Mateu and Solé, Spain). The process was carried out at injection and maintaining pressures of 120 and 37.5 kg/cm^3^, respectively, and temperatures of 175, 175, and 190 °C for the three equipment heating areas. At least 10 flexural specimens of each formulation were obtained to be posteriorly tested. The dimensions of the obtained specimens were around 127 mm in length, 13.2 mm in width, and 3.1 mm in thickness.

Before the flexural tests, the specimens were stored in a conditioning chamber for 48 h, 23 °C, and 50% relative humidity, as required by [29]. Then, the specimens were subjected to a three-point bending test using an Instron universal testing machine fitted with a 5 kN load cell, following [30]. Some important specifications about the three-point bending test include a distance between supports of 50 mm and 2 mm/min testing speed.

The average and standard deviation of the flexural modulus and maximum flexural deformation were taken from 10 sample tests.

#### 2.2.3. Morphological Analysis of the Fibers

The morphology of the fibers is required to use some micromechanics models. Since the compounding and injection molding processes change the fiber’s morphology, it is necessary to evaluate its morphology after the whole compounding process and not before. For this reason, the cotton fibers were recovered from the composite materials by PP solubilization using a Soxhlet apparatus by Merck KGaA (Dramstadt, Germany) and decalin (decahydronaphthalene) as solvent. The whole extraction process lasted about 24 h, and then the fibers were rinsed with acetone and distilled water to remove the remaining solvent. The morphology of the fibers was evaluated using a MorFi Compact from Techpap SAS (Gières, France). The equipment measures about 30,000 fibers per test and, amongst other parameters, returns the average fiber lengths and diameters of the fibers.

Scanning electron microscopy (SEM) was performed on the cross-sectional area of the fractured specimens using a Zeiss DMS 960 SEM microscope by Zeiss (Jena, Germany).

#### 2.2.4. Density Measurement and Void Volume Percentage

The density of the composite ($\rho^{c}$) and matrix ($\rho^{m}$) was determined using a pycnometer and distilled water as reference liquid. Then, the density of the fibers ($\rho^{F}$) was obtained from Equation (1), whereas the fiber volume fraction ($V^{F}$) was calculated following Equation (2):

(1) $$\rho^{C}=\frac{w^{C}}{w^{m}/\rho^{m}+w^{F}/\rho^{F}}\;$$

(2) $$V^{F}=\frac{w^{F}/\rho^{F}}{w^{F}/\rho^{F}+w^{m}/\rho^{m}}$$

where $w^{C}$, $w^{m},$ and $w^{F}$ represent the composite, matrix, and fiber weight fractions, respectively. Void volume percentage ($V_{void})$ was estimated using the expression in Equation (3) [31]:

(3) $$V_{void}=\left(\frac{\rho^{C}{}_{th}-\rho^{C}{}_{ex}}{\rho^{C}{}_{th}}\right)\cdot 100$$

where $\rho^{C}{}_{th}$ and $\rho^{C}{}_{ex}$ are the theoretical and experimental density of the composites. The theoretical density of the composites is calculated assuming a fiber density of 1.54 g/cm^3^, which agrees with accepted values in the literature.

#### 2.2.5. Modeling the Flexural Modulus

Modeling the behavior of natural fiber composites is often required to gain a deeper understanding of the reinforcing mechanisms and to take advantage of the potential of the composites. Additionally, natural fiber-reinforced composites exhibit complex behavior under load due to their anisotropy, which further supports the use of such micromechanics models. One of the simplest ways to compute the contribution of the fibers and matrix to the flexural modulus of the composite is through a modified Rule of Mixtures (mRoM). The rule was initially developed to model Young’s modulus [30], although it was rapidly adapted to the flexural modulus [32]. The mRoM for the flexural modulus is presented in Equation (4):

(4) $$E_{f}^{C}=\eta_{e}\cdot E_{f}^{F}\cdot V^{F}+E_{f}^{M}\cdot \left(1-V^{F}\right)$$

where $E_{f}^{C}$, $E_{f}^{F}$, and $E_{f}^{M}$ are the flexural modulus of the composite, fiber, and matrix, respectively. The fiber volume fraction is represented by $V^{F}$, whereas the contribution of the fibers to the flexural modulus of the composite is corrected by incorporating a modulus efficiency factor ($\eta_{e}$). The fiber volume fraction may be simply calculated from the density of the composite ($\rho^{C}$) and the polymer ($\rho^{M}$), both measured using a pycnometer, and from the fiber ($w^{F}$) and matrix ($w^{M}$) weight fractions [33].

The contribution of the fibers to the overall flexural modulus of the composite can be determined from a Fiber Flexural Modulus Factor (FFMF) by rearranging the mRoM. The contribution of the fibers to the flexural modulus of the composite, expressed by $E_{f}^{C}-E_{f}^{M}\cdot \left(1-V^{F}\right)$, is graphically represented as a function of the fiber volume fraction ($V^{F}$) at each fiber content (Equation (5)). The FFMF is obtained from the slope of the regression line that joins the contributions at different reinforcement volume fractions. The parameter has been typically used in the literature for comparison purposes with other types of reinforcement within the same matrix.

(5) $$\mathrm{FFMF}=\eta_{e}\cdot E_{f}^{F}=\frac{E_{f}^{C}-E_{f}^{M}\cdot \left(1-V^{F}\right)}{V^{F}}\;$$

The mRoM, in its current shape, contains two unknowns, which are the intrinsic flexural modulus of the fibers ($E_{f}^{F}$) and modulus efficiency factor ($\eta_{e}$). Following previously published methodologies, the intrinsic flexural modulus can be effectively determined using either (i) the Hirsch model [34] or (ii) the Tsai–Pagano model employing Halpin–Tsai equations, hereby abbreviated as TP&HT [35,36,37].

The Hirsch model is a combination of Reuss and Voigt models. The Reuss model defines a system where the load is applied parallel to the fiber axis, whereas in the Voigt model, the stress happens perpendicular to the fiber axis. From the combination of both models and, by the inclusion of a stress-transfer coefficient (β), the Hirsch model is obtained. Generally, in those short fiber polymer composites processed using injection molding, a value of β close to 0.4 has shown good agreement between theoretical and experimental values [38]. The Hirsch model is reported in Equation (6):

(6) $$E_{f}^{c}=\beta \cdot \left(E_{f}^{F}\cdot V^{F}-E_{f}^{M}\left(1-V^{F}\right)\right)+\left(1-V^{F}\right)\cdot \frac{E_{f}^{F}\cdot E_{f}^{M}}{E_{f}^{M}\cdot V^{F}+E_{f}^{F}\cdot \left(1-V^{F}\right)}$$

Unlike the Hirsch model, where only experimental data from the flexural test are used, the TP and HT model also considers morphological features of the fibers, such as mean fiber length ($l^{F}$) and diameter ($d^{F}$). Tsai–Pagano model is described in Equation (7), whereas the longitudinal modulus ($E^{11}$) and transverse modulus ($E^{22}$) may be determined following Halpin–Tsai equations according to Equations (8) and (9):

(7) $$E_{f}^{C}=\frac{3}{8}\;\cdot E^{11}+\frac{5}{8}\;\cdot E^{22}$$

(8) $$E^{11}=\frac{1+2\cdot \left(l^{F}/d^{F}\right)\cdot \eta_{l}\cdot V^{F}\;}{1-\eta_{l}\cdot V^{F}}\cdot E_{f}^{m}\;;\;\;\eta_{l}=\frac{\left(E_{f}^{F}/E_{f}^{M}\right)-1}{\left(E_{f}^{F}/E_{f}^{M}\right)+2\cdot \left(l^{F}/d^{F}\right)}\;\;\;$$

(9) $$E^{22}=\frac{1+2\cdot \eta_{t}\cdot V^{F}\;}{1-\eta_{t}\cdot V^{F}}\cdot E_{f}^{m}\;;\;\;\eta_{t}=\frac{\left(E_{f}^{F}/E_{f}^{M}\right)-1}{\left(E_{f}^{F}/E_{f}^{M}\right)+2}$$

Once the intrinsic flexural modulus is computed, either by Hirsch or TP&HT models, the modulus efficiency factor may be obtained from the mRoM in Equation (4). Such modulus efficiency factor is mainly influenced by the orientation and length of the fibers inside the composite, which makes it possible to decompose the factor in a modulus orientation factor ($\eta_{l}$) and modulus length factor ($\eta_{o}$) according to Equation (10):

(10) $$\eta_{e}=\eta_{l}\cdot \eta_{o}$$

Both $\eta_{l}$ and $\eta_{o}$ can be obtained by initially calculating the $\eta_{l}$ through the Cox and Krenchel model (Equation (11)) [39,40] and then isolating the $\eta_{o}$ from Equation (10). It should be noted that the factor ξ in Equation (11) refers to the stress concentration rate at the ends of the fibers, whereas ν is the Poisson’s ratio of the matrix—in this case, 0.36 for PP.

(11) $$\eta_{l}=1-\frac{\tan h\;\left(\xi \cdot l^{F}/2\right)}{\xi \cdot l^{F}/2}\;;\;\;\xi =\frac{1}{\left(d^{F}/2\right)}\cdot \sqrt{\frac{E_{f}^{M}}{E_{f}^{F}\cdot \left(1-\nu \right)\cdot \sqrt{\ln(\pi /4V^{F})}}}$$

Once the orientation factor is obtained from Equation (10), it is possible to relate such factor to a limiting angle of the fibers ($\alpha_{o}$) following the Fukuda and Kawata model (Equation (12)) [41]. Then, Sanomura and Kawamura [42] proposed an orientation parameter ($f_{p}$) from which a theoretical average orientation of the fibers (α) can be obtained ((Equation (13)):

(12) $$\eta_{o}=\frac{\sin\left(\alpha_{o}\right)}{\alpha_{o}}\cdot \left(\frac{3-\nu }{4}\cdot \frac{\sin\left(\alpha_{o}\right)}{\alpha_{o}}+\frac{1-\nu }{4}\cdot \frac{\sin\left(3\alpha_{o}\right)}{3\alpha_{o}}\right)$$

(13) $$f_{p}=\frac{\sin\left(2\alpha_{o}\right)}{2\alpha_{o}}=2\cdot \cos^{2}\left(\alpha \right)-1$$

**Figure 1:**
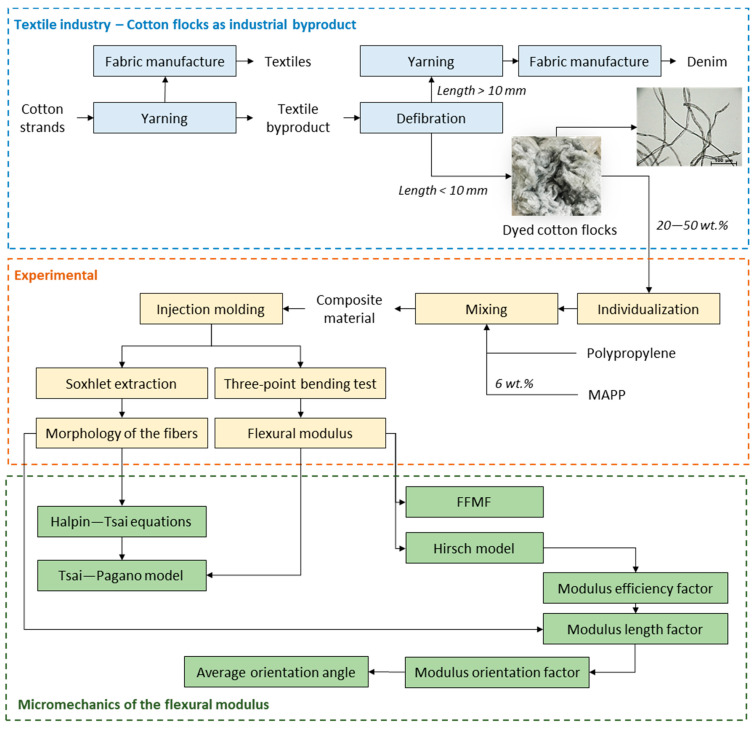
Workflow of the present investigation.

The workflow of the current investigation, from experimental to micromechanics modeling, is presented in Figure 1.

## 3. Results

### 3.1. Density and Void Volume Percentage of the Composites

The effect of fiber loading on the density and void volume percentage of the composites was evaluated and the results are collected in Table 1.

The density of the composites is observed to increase with the fiber content due to the notably higher density of cotton fibers in comparison with the neat matrix. In this sense, the density of cotton fibers was calculated to be 1.50 g/cm^3^, assuming fully dense composite materials. However, the reported density in the literature for cotton fibers has been set at 1.54 g/cm^3^, from which the theoretical density of the composite can be back calculated with the purpose of obtaining the void volume percentage of the composites (Equation (3)). Accordingly, the manufactured composites yielded void volume percentages of 0.339, 0.460, 0.715, and 0.881% concerning the 20, 30, 40, and 50 wt.% fiber content.

Porosity or void content in natural fiber composites is usually due to the intra fiber voids, such as natural fiber lumen, or due to the formation of voids between fibers. The first phenomenon affects composites with low fiber content, and the second increases with the percentage of fibers, uneven dispersion, or low individualization. Indeed, it is observed that void content increased with the fiber volume fraction. In this work, the relatively low void volume percentages are principally attributed to the compression forces that the materials undergo due to the mold injection process. Fibers’ morphology changes noticeably during this phase. The lumen disappears as the fibers are compressed. On the other hand, the authors individualized the fibers prior to their processing to minimize the apparition of fiber bundles and to ensure a proper and regular dispersion of the reinforcements in the composite. This fact decreases the formation of voids between fibers.

Voids may affect the mechanical performance of composite materials. Madsen et al. [43] stated that for natural fiber-reinforced thermoplastic composites, the effect of porosity on the stiffness could be approximated by a factor of ${\left(1-V_{void}\right)}^{2}$. This roughly means that the void content in the composites reduced the stiffening potential by 0.68, 0.92, 1.42, and 1.75% concerning the composites containing a 20, 30, 40, and 50 wt.% of cotton fibers. These are considered almost negligible effects. Indeed, it has been considered that void content up to 4% has minimal effect on natural fiber composites [44]. Neglecting the possible effects of porosity on the materials’ stiffness, the flexural modulus of the manufactured composites was evaluated.

### 3.2. Analysis of the Flexural Modulus

**Figure 2:**
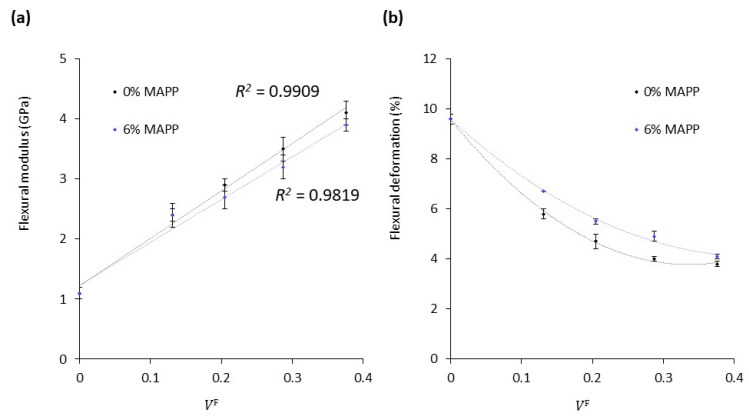
Evolution of the (**a**) flexural modulus and (**b**) flexural deformation with the fiber volume fraction of the composites against reinforcement volume fraction.

Table 2 presents the flexural modulus ($E_{f}^{C}$) and flexural strain ($\varepsilon_{f}^{C}$) of the coupled (6 wt.% MAPP) and uncoupled (0 wt.% MAPP) polypropylene (PP) composites containing 20–50 wt.% of cotton fibers (CF). In addition, the evolution of such parameters with the fiber volume fraction is graphically represented in Figure 2.

It is observed in Table 2 that the flexural modulus of the composites increased noticeably with the fiber content from 1.1 GPa, corresponding to neat PP, to 4.1 and 3.9 GPa (uncoupled and coupled materials, respectively) at 50 wt.% of CF. The increments in the flexural moduli were linearly correlated with the fiber contents as reflected in the high, close to 1, linear correlation coefficients (R^2^) in Figure 2a. Such linear increase between both variables has been reported to be an indicator of good dispersion of the fibers within the polymeric phase [33]. This behavior contrasts with other studies reporting that lignocellulosic fibers, especially those with high cellulose contents, tend to aggregate when combined with hydrophobic polymers such as PP or PE, ultimately reducing or hindering the increment of the flexural modulus [45]. The literature suggests that optimum fiber content is found between 15 and 25 wt.% for polyolefin or polyester-based composites [46,47,48]. Hence, keeping a good dispersion of the phases at elevated fiber contents is considered relevant to effectively replace part of the plastic material with natural fibers, contributing to cost reductions and environmental impact reductions.

Since cotton is a high cellulose content material, precisely 93.8 wt.% of cellulose and 0.5 wt.% of lignin [23], its good dispersion inside the composite material can be presumably attributed to the presence of dyes. Such dyes, which are also a residue in the industrial production of denim products, can act as hydrophobic agents of the fiber surface and thus improve the fiber-matrix compatibility. As it was reported in a previous study, the cationic demand (CD) of the dyed cotton flocks was 16.39 μeq·g/g, whereas the virgin cotton fibers containing no dyes exhibited a CD of about 58.7 μeq·g/g [23,49]. This means that the presence of dyes reduces the anionic nature of the fiber surface and thus makes the reinforcement more affine to the plastic material. For this reason, the presence of dyes in cotton flocks should not be viewed as inconvenient if the purpose is to valorize the residue in the composite sector. 

**Figure 3:**
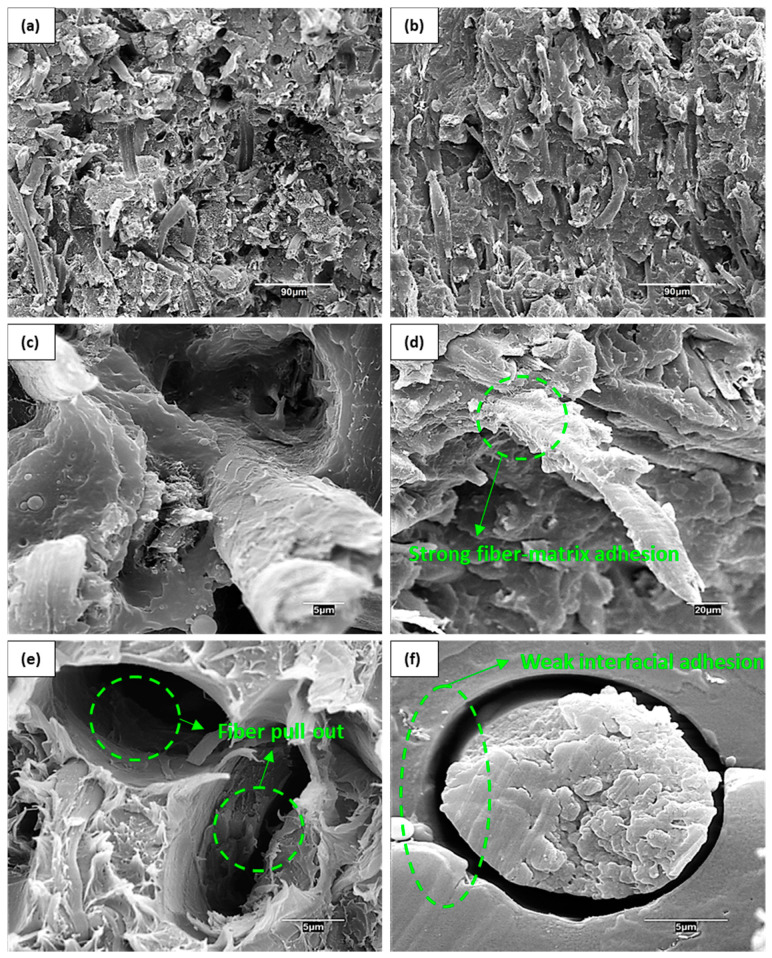
SEM micrographs at the cross-sectional area of the flexural specimens in composites containing 40 wt.% of cotton fibers. Observation of the composites with (**a**) and without (**b**) MAPP at low magnifications. Observation at higher magnification of composites with MAPP (**c**,**d**) and without MAPP (**e**,**f**).

The differences in flexural moduli of both coupled and uncoupled composites were not statistically significant as determined by ANOVA analysis at 95% confidence. Indeed, the presence of coupling agents that enhance the fiber-matrix interfacial adhesion has been reported to have almost no influence on the stiffness of composite materials [48], which is mainly governed by other factors, such as fiber and matrix properties, fiber content, grade of dispersion, and distribution of the phases [26,50,51]. Hence, coupling agents such as MAPP may not be required for purely stiffening purposes. However, composites containing MAPP were able to withstand higher flexural deformations and in addition, a recent study indicated that the flexural strength of coupled composites was higher than uncoupled [52]. Contrary to the flexural modulus, the analysis of variance (ANOVA) for the flexural deformation revealed significant differences between coupled and uncoupled composites. Such effects on deformation and strength properties are explained by the presence of MAPP favoring the bond formation between PP and cotton fibers, which consequently improves the stress transfer at the interfacial boundary when the material is subjected to load. Additionally, MAPP may also contribute to the water barrier properties of the composite material by reducing the availability of hydroxyl groups [53]. It is concluded that MAPP may not be necessary to increase the flexural modulus of the composite, though, its addition may add a competitive advantage over uncoupled composites in terms of strength, deformation capacity, and water barrier properties. Scanning electron microscopy (SEM) images were taken at the cross-sectional area of the specimens to assess how the presence of MAPP affected the dispersion and adhesion of the phases (Figure 3).

In Figure 3a, several holes in the material can be observed, presumably due to fiber slippage because of poor fiber-matrix adhesion in those uncoupled composites. A rather smother surface is observed in Figure 3b for the coupled composite, and the fibers seem to be more attached to the matrix. Such effects are more pronounced at higher magnifications. Composites without MAPP (Figure 3c,d) showed weaker interfacial adhesion than those containing MAPP (Figure 3e,f), as denotes the poor fiber-matrix anchoring and fiber pull-out tendency. Moreover, coupled composites showed improved wetting of the polymer on the fiber mainly due to the improved interactions between both phases. These effects on the material can explain the lower deformation capacities of uncoupled composites over coupled ones.

### 3.3. Contribution of Cotton Fibers to the Flexural Modulus of the Composites

**Figure 4:**
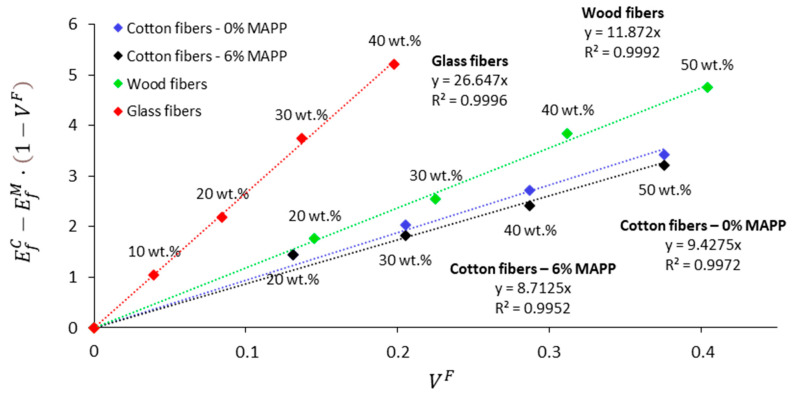
Representation of the Fiber Flexural Modulus Factor (FFMF) of cotton fibers, wood fibers, and glass fibers as PP reinforcement.

The Fiber Flexural Modulus Factor (FFMF) is used to compute the contribution of the fibers to the flexural modulus of the composite. The parameter is considered an adequate indicator of the stiffening potential of natural fibers and thus can be used for comparison purposes with other fibers. In this work, the FFMF of cotton fibers was compared with other PP-based composites reinforced with wood fibers [54] and glass fibers [55] and reported in the literature. In Figure 4, the net contribution of cotton fibers, wood fibers, and glass fibers to the flexural modulus of PP composites is represented against the fiber volume fraction to obtain the FFMF. For comparison purposes, the fiber weight percentage is also presented in Figure 4.

The FFMFs of cotton fiber uncoupled and coupled composites were 9.43 and 8.71, respectively. The FFMFs of wood fibers and glass fibers reinforced PP were 11.87 and 26.65, respectively. This roughly means that with equal increases in the fiber volume fraction, wood fibers and glass fibers are expected to have a stiffening effect of about 1.3 and 2.9 times higher than cotton fibers. From Figure 4 it is further possible to estimate that, for instance, 40 wt.% of cotton fibers, 30 wt.% of wood fibers, and 20 wt.% of glass fibers showed similar stiffening potential. It is also worth noting that at the mentioned weight percentages, cotton fiber composites show rather higher flexural deformation than wood composites (4.7%), which can be principally attributed to a better grade of dispersion within the matrix. Other types of lignocellulosic residues resulting from the textile industrial activity have shown slightly lower FFMFs. This is the case of hemp core fibers, which are an industrial residue from the production of hemp strands, which when combined with PP exhibited an FFMF around 8 [56].

Following the same methodology as for the calculus of the FFMF, a Fiber Tensile Modulus Factor (FTMF) can be calculated from the measurement of Young’s modulus. The ratio between the FFMF and FTMF has been proposed in recent works as a simple modus for predicting the intrinsic flexural modulus of natural fibers [19]. Such a hypothesis is built on the fact that the modulus efficiency factor ($\eta_{e}$) does not depend upon the type of test, either tensile or flexural. $\eta_{e}$ is mainly influenced by fibers’ morphology, dispersion, and average orientation within the composite material, and such characteristics should not vary with the type of test. Assuming this hypothesis is correct, the following expression is obtained (Equation (14)):

(14) $$\frac{\mathrm{FFMF}}{\mathrm{FTMF}}=\frac{\eta_{e}\cdot E_{f}^{F}}{\eta_{e}\cdot E_{t}^{F}}=\frac{E_{f}^{F}}{E_{t}^{F}}\;\;\;\;\;\;\mathrm{then},\;\;\;\;\;\;E_{f}^{F}=\frac{\mathrm{FFMF}}{\mathrm{FTMF}}\cdot \;E_{t}^{F}$$

The FTMF and intrinsic tensile modulus of cotton fibers ($E_{t}^{F}$) were studied in previous work [9]. The FTMF was set at 12.603, whereas the $E_{t}^{F}$ was 31.5, 28.1, 26.5, and 25.5 GPa with respect to the composites containing 20, 30, 40, and 50 wt.% of cotton fibers, respectively. From these values, the intrinsic flexural strength of cotton fibers may be obtained following Equation (14). It is noted that the resulting values should be further contrasted with more established models such as Hirsch or Tsai–Pagano ones. However, finding models that connect, in a rather effective way, tensile and flexural properties of composite materials is interesting from a research perspective, as reflected in several works, especially in the study of fibers’ intrinsic properties owing to the difficulty of direct measuring their properties [57,58]. For instance, Hashemi [59] proposed a linear relationship combining macro- and micromechanical parameters by using the ratio between the tensile and flexural modulus of the composite, resulting in the following expression: $E_{f}^{F}=\left(E_{f}^{c}/E_{t}^{c}\right)\cdot \;E_{t}^{F}$. The main difference between this expression and the one in Equation (14) is that the latter one accounts only for the fiber contribution, whereas the expression of Hashemi considers both the matrix and fiber contribution to the flexural modulus of the composite.

### 3.4. Determination of the Intrinsic Flexural Modulus

**Figure 5:**
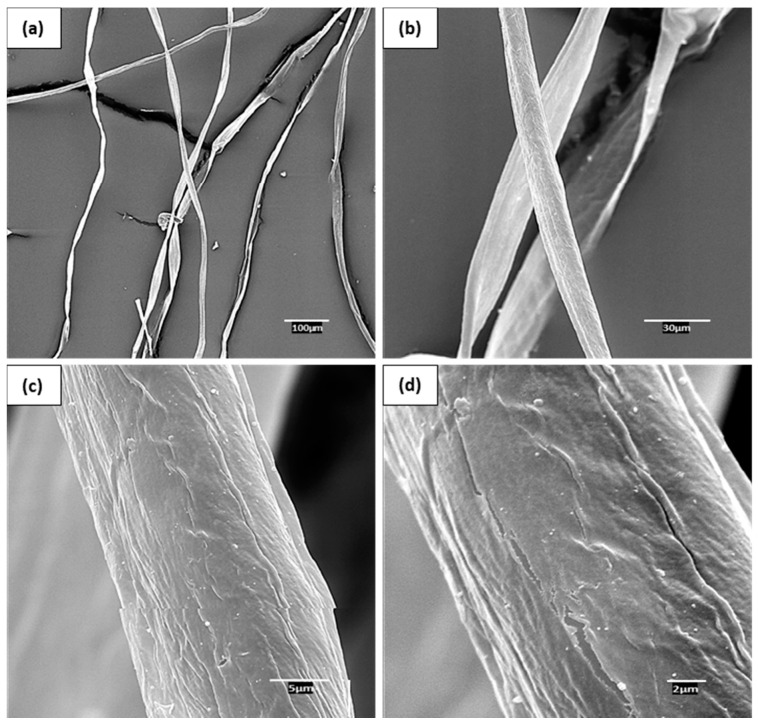
SEM images of cotton fibers before processing at different magnifications: (**a**) Cotton fibers at 100 µm of magnification; (**b**) Cotton fibers at 30 µm of magnification; (**c**) Cotton fiber at 5 µm of magnification; (**d**) Cotton fiber at 2 µm of magnification.

The difficulty in measuring the flexural modulus of natural fibers by direct testing glimpses the opportunity of applying micromechanics models. In this context, the intrinsic flexural modulus of cotton fibers was calculated using (i) the Hirsch model and (ii) the Tsai–Pagano model using Halpin–Tsai equations, abbreviated as TP&HT. Since Halpin–Tsai equations require the mean fiber length ($l^{F}$) and diameter ($d^{F}$) of the fibers, the composite materials were subjected to Soxhlet extraction using decalin as a solvent to dissolve polypropylene and recover the fibers, which were then submitted to morphological analysis. The results from the morphological test are reported in Table 3. Further, SEM images of the cotton fibers before processing are provided in Figure 5 to further evaluate the morphology of the fibers.

A clear overall tendency of the mean fiber length to decrease as the fiber content increases was observed. This is explained by an increment of the composite viscosity concerning the matrix when natural fibers are incorporated into the material, which by consequence increases the shear forces created during the internal mixing process leading to fiber attrition and fiber length shortening. Additionally, the diameter was less affected by the compounding process and thus remained very stable at 16.5 µm. Such diameter agrees with the images in Figure 5, where the fibers present approximate diameters in the range of 16 and 17 µm. In addition, from SEM images in Figure 5, one can see that cotton fibers present a smooth surface, probably due to low lignin content and to the presence of dyes covering the surface. The attrition phenomena of the fibers were more pronounced in coupled composites, as reflected in a more sudden decrease of the average fiber length. This is explained by the stronger fiber-matrix adhesion in coupled composites that promotes the more efficient transmission of the shear forces from the matrix to the fibers [60]. The lower length of natural fibers in coupled composites, combined with very stable diameters, also led to smaller aspect ratios. Such differences observed in the aspect ratio of the fibers depending on the presence of MAPP can justify the slight discrepancies in the flexural moduli between uncoupled and coupled composites. The relationship between fiber aspect ratio and the stiffness of composite materials has been previously studied by Shibata et al. (2005) [61] and Hsueh (2000) [62]. According to their results, the stiffness of composites increases with the average fiber length up to approximately 2.8 mm length. Hence, the uncoupled composites are expected to show slightly higher flexural modulus owing to the superior aspect ratios of the fibers. This also justifies the somewhat higher FFMF of uncoupled composites.

Both average fiber length and diameter were incorporated into Halpin–Tsai equation. The intrinsic flexural modulus of cotton fibers was determined following three different routes being (i) the Hirsch model, (ii) the TP&HT model, and (iii) the one proposed in this work using the FFMF/FTMF ratio. The results are presented in Table 4.

**Figure 6:**
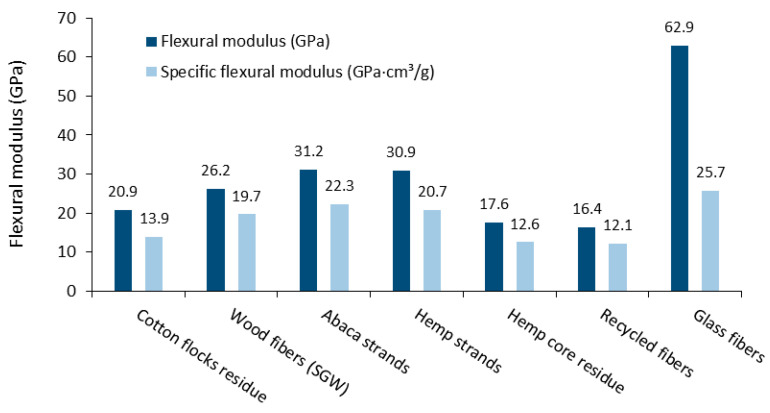
Flexural modulus and specific flexural modulus of different fiber-reinforced PP composites (wood fibers [52], abaca strands [53], hemp strands [61], hemp core residues [54], recycled fibers [32], and glass fibers [53]).

The different models applied for the calculus of the intrinsic flexural modulus showed good agreement, especially above the 20 wt.% fiber content. The similarities between methodologies support the utility of the Hirsch model, as well as the approach proposed in this work by using the FFMF/FTMF ratio, in front of Tsai–Pagano model, because these models do not require morphological data. It is further observed that the intrinsic flexural moduli of the fibers tended to decrease as the fiber content was increased. This could be due to fiber attrition phenomena suffered during compounding, which is intensified at higher fiber contents. Assuming the values obtained from the Hirsch model as being accurate, the intrinsic flexural modulus of cotton fibers was compared with other fiber-reinforced PP composites, as shown in Figure 6. The specific intrinsic flexural modulus ($E_{f}^{F}/\rho^{F}$) of the fibers are also included in Figure 6 to attain the different densities of the fibers.

The intrinsic flexural modulus of cotton fibers was higher than other lignocellulosic residues, such as hemp core and recycled fibers, though still slightly below those of wood fibers and strands from annual plants such as abaca or hemp. The intrinsic flexural modulus of glass fibers was notably higher than those of natural fibers, though its high density (2.45 g/cm^3^) makes the specific property comparable with the others.

### 3.5. Modulus Efficiency, Length, and Orientation Factors

The modulus efficiency factor was computed considering the intrinsic flexural strength of cotton fibers and the modified Rule of Mixtures (mRoM). The implication of fiber morphology and orientation on the flexural modulus of the composite was evaluated using the modulus length and orientation factors. Further, from the orientation factor, the limiting and average orientation angles were determined. The results are given in Table 5.

First, the slight differences between the micromechanics of coupled and uncoupled composites are not considered significant, as the variations are almost negligible. The modulus efficiency factor is in line with those reported in the literature for natural fibers, around 0.5, supporting the relevance of the results and indicating that the composite material took full advantage of the stiffening potential of the fiber. Noteworthily, the efficiency factor is in the same order as the one obtained from the micromechanics of Young’s modulus in a recent work, with an average value of 0.47. The similarities between the efficiency factors obtained from Young’s and flexural moduli analyses support the theoretical hypothesis regarding the calculus of the intrinsic flexural modulus from the FFMF/FTMF ratio.

The modulus orientation factor typically ranges between 0.4 and 0.6 in composites processed using injection molding and is mainly influenced by the compounding conditions and mold geometry [46]. In this work, the values for the modulus orientation factor were around 0.53–0.54. Such factor equals 1 when the fibers are completely aligned, whereas for planar random configuration the factor tends to be 3/8 and for completely random systems the factor may decrease to 1/5 [63,64]. Hence, for the present case a certain alignment of the fibers inside the composite is noticed, which as mentioned, can be principally attributed to the injection process. The modulus length factor was close to 1, which indicates that fibers’ morphology, particularly their aspect ratio, plays a key role in stiffening the composite material by providing an adequate load transfer.

By using the modulus orientation factor and estimating a square packing distribution of the fibers inside the composite, a limiting angle around 63–64° was obtained. The limiting angle refers to the angle of orientation for which the axial stress of the fiber tends to 0. From such value, the mean orientation of the fibers inside the composite may be set at 30.6°, both for uncoupled and coupled composites, considering that a fully aligned fiber/matrix system would reach 0°. Comparatively, the micromechanics of Young’s modulus for the same composites returned a limiting angle of 53.3° and mean orientation about 36.1°. It is observed that, again, the micromechanics of the Young’s modulus and flexural modulus deliver similar results. Similarities are also found in the micromechanics analysis of the tensile strength, which was the subject of study in previous work [24]. In such work, the strength orientation factor ($x_{1}$) was set at 0.3, from which a limiting angle of 61° could be calculated in agreement with the expression $x_{1}=\cos^{4}\left(\alpha_{0}\right)$ developed by Mittal et al. [65], finally obtaining a mean orientation of the fibers of 35.4°. It is observed that from tensile strength, Young’s modulus, and flexural modulus analysis of the materials’ similar orientation angles are attained, which supports the relevance of the micromechanics models and the results obtained.

In general, the developed cotton-reinforced PP composites are presented as an attractive opportunity to replace virgin lignocellulosic feedstocks with industrial residues, attaining economically and technically competitive materials. The composites could exhibit potential for semi-structural/construction given their relatively high stiffness/weight ratio, making their use particularly adequate for lightweight applications, such as panels, ceilings, and partition boards. Other fields where the composites could be directly implemented concern the automotive sector. Indeed, the global production of natural fiber composites in the automotive sector has grown considerably in recent years from 90,000 tons in the year 2010 to 350,000 tons by 2020 [66]. Additionally, the similar appearance of the composites to solely wood materials also makes the composites suitable for furniture and indoor applications.

## 4. Conclusions

The textile industry generates huge amounts of cotton byproducts yearly, from which only a minor part is recycled, and the rest is either landfilled or incinerated, resulting in serious environmental issues. To enhance the sustainability of the textile sector, the potential of these cotton byproducts in the composite sector was evaluated. The cotton flocks were mechanically defibrated and incorporated into a polypropylene matrix up to 50 wt.%. The flexural modulus of the composites was studied to assess their potential and competitiveness. The results indicate that the flexural modulus increased steadily up to 50 wt.% of fiber contents, increasing the flexural modulus of the matrix by 272%. The good dispersion of the phases in comparison with other fiber typologies was attributed to the presence of dyes, whereas the good interfacial adhesion was attributed to the presence of maleic anhydride polypropylene (MAPP), which was used as a coupling agent. From the micromechanics analysis of the flexural modulus, the following remarks are pointed out: (i) An alternative methodology, which connected Young’s and flexural moduli, was used for the calculus of the intrinsic flexural modulus of cotton fibers, which agreed with more established models such as Hirsch or Tsai–Pagano; (ii) The intrinsic flexural modulus (around 20 GPa) was found in line with other natural fibers, though still far from glass fibers; (iii) It was shown that the composite took advantage of the stiffening potential of cotton fibers; (iv) The fibers showed a certain degree of alignment, whereas the aspect ratio was found to play a key role on stiffening the material. Overall, the developed composites showed similar stiffening potential as other natural fibers, with the environmental and economic advantage of valorizing a low value-added residue.

## Figures and Tables

**Table 1:** Fiber content, matrix density ($\rho^{m}$), experimental composite density ($\rho^{C}{}_{ex}$), fiber density ($\rho^{F}$), fiber volume fraction ($V^{F}$), theoretical composite density ($\rho^{C}{}_{th}$), and void volume percentage ($V_{void}$).

Fiber Content (wt. %)	$\rho^{m}\;(g/{\mathrm{cm}}^{3})$	$\rho^{C}{}_{ex}\;(g/{\mathrm{cm}}^{3})$	$\rho^{F}\;(g/{\mathrm{cm}}^{3})$	$V^{F}$	$\rho^{C}{}_{th}\;(g/{\mathrm{cm}}^{3})$	$V_{void}\;(\%)$
20	0.905	0.983	1.500	0.131	0.986	0.339
30		1.028	1.505	0.205	1.033	0.460
40		1.076	1.502	0.287	1.084	0.715
50		1.129	1.504	0.376	1.140	0.881

**Table 2:** Flexural modulus ($E_{f}^{C}$) and flexural deformation ($\varepsilon_{f}^{C}$) of the uncoupled and coupled composites reinforced with cotton fibers.

**Fiber Content** **(wt. %)**	**V^f^**	0 wt.% MAPP		6 wt.% MAPP	
		$E_{f}^{c}$ (GPa)	$\varepsilon_{f}^{c}$ (%)	$E_{f}^{c}$ (GPa)	$\varepsilon_{f}^{c}$ (%)
0	0	1.1 ± 0.1	9.6 ± 0.2	1.1 ± 0.1	9.6 ± 0.2
20	0.131	2.4 ± 0.2	5.8 ± 0.2	2.4 ± 0.1	6.7 ± 0.0
30	0.205	2.9 ± 0.1	4.7 ± 0.3	2.7 ± 0.2	5.5 ± 0.1
40	0.287	3.5 ± 0.2	4.0 ± 0.1	3.2 ± 0.2	4.9 ± 0.2
50	0.376	4.1 ± 0.2	3.8 ± 0.1	3.9 ± 0.1	4.1 ± 0.1

**Table 3:** Evolution of morphological parameters, mean fiber length ($l^{F}$), mean fiber diameter ($d^{F}$), and aspect ratio ($l^{F}/d^{F}$) with the fiber content.

Fiber Content (wt. %)	0 wt.% MAPP			6 wt.% MAPP		
	$l^{F}\;$ **(µm)**	$d^{F}$ **(µm)**	$l^{F}/d^{F}$	$l^{F}$ **(µm)**	$d^{F}$ **(µm)**	$l^{F}/d^{F}$
20	512 ± 9	16.5	31.0	509 ± 9	16.5	30.8
30	459 ± 5		27.8	406 ± 5		24.6
40	396 ± 13		24.0	339 ± 7		20.5
50	351 ± 15		21.3	299 ± 11		18.1

**Table 4:** Intrinsic flexural modulus of cotton fibers obtained using (i) Hirsch model, (ii) Tsai–Pagano model using Halpin–Tsai equation (TP&HT) (iii), and the ratio between the FFMF and FTMF.

Fiber Content (wt. %)	Intrinsic Flexural Modulus $\left(E_{f}^{F}\right)$ (Gpa)					
	0 wt.% MAPP			6 wt.% MAPP		
	Hirsch	TP&HT	$\frac{FFMF}{FTMF}$	Hirsch	TP&HT	$\frac{FFMF}{FTMF}$
20	24.1	29.4	21.8	24.1	29.4	23.3
30	21.1	23.7	19.4	18.7	20.4	20.8
40	19.9	21.4	18.3	17.3	18.1	19.6
50	18.6	18.9	17.6	17.3	17.6	17.6
Average	20.9	23.4	20.7	19.4	21.4	19.3

**Table 5:** Micromechanics of the flexural modulus. Intrinsic flexural strength ($E_{f}^{F}$), modulus efficiency factor ($\eta_{e}$), length factor ($\eta_{l}$), orientation factor ($\eta_{o}$), limit angle ($\alpha_{o}$), mean orientation angle (α).

Coupling Agent Content	Factor	Fiber Content (wt. %)				Average
		20	30	40	50	
**0 wt.% MAPP**	$E_{f}^{F}$ (Hirsch)	24.1	21.1	19.9	18.6	20.9
	$\eta_{e}$	0.46	0.47	0.48	0.49	0.47
	$\eta_{l}$	0.89	0.90	0.90	0.91	0.90
	$\eta_{o}$	0.52	0.52	0.53	0.54	0.53
	$\alpha_{o}$	66.0°	65.2°	64.3°	63.1°	64.7°
	α	30.6°	30.6°	30.6°	30.6°	30.6°
**6 wt.% MAPP**	$E_{f}^{F}$ (Hirsch)	24.1	18.7	17.3	17.3	19.4
	$\eta_{e}$	0.46	0.48	0.49	0.49	0.48
	$\eta_{l}$	0.89	0.89	0.89	0.89	0.89
	$\eta_{o}$	0.52	0.54	0.55	0.55	0.54
	$\alpha_{o}$	65.8°	63.5°	61.9°	61.2°	63.1°
	α	30.6°	30.6°	30.6°	30.6°	30.6°
